# Aberrant methylation-mediated downregulation of lncRNA SSTR5-AS1 promotes progression and metastasis of laryngeal squamous cell carcinoma

**DOI:** 10.1186/s13072-019-0283-8

**Published:** 2019-06-13

**Authors:** Baoshan Wang, Lei Zhao, Weiwei Chi, Huan Cao, Weina Cui, Wenxia Meng

**Affiliations:** 1 Otolaryngology Head and Neck Surgery Department, The Second Hospital of Hebei Medical University, Heping West Road 215, Shijiazhuang, 050005 Hebei China; 2 Otolaryngology Head and Neck Surgery Department, The First Hospital of Hebei Medical University, Shijiazhuang, Hebei China

**Keywords:** SSTR5, SSTR5-AS1, Laryngeal squamous cell carcinoma, Expression, Methylation

## Abstract

**Background:**

Laryngeal squamous cell carcinoma (LSCC) is among the most common malignant tumors with poor prognosis. Accumulating evidences have identified the important roles of long noncoding RNAs (lncRNAs) in the initiation and progression of various cancer types; however, the global lncRNAs expression profile for metastatic LSCC is limited.

**Results:**

In the present study, we screen expression profiles of lncRNAs in advanced LSCC patients with paired tumor tissues and corresponding normal tissues by microarrays. We identify numerous differentially expressed transcripts, and after the necessary verification of the transcripts expression in expanded samples, we experimentally validate the expression patterns of the remarkable low expressed gene, SSTR5, and its antisense lncRNA, SSTR5-AS1. Downregulation of SSTR5 is detected in LSCC tissues and laryngeal carcinoma cells. Aberrant DNA hypermethylation of the CpG sites clustered in the exon 1 and accumulation of inactive histone modifications at SSTR5 promoter region may be epigenetic mechanisms for its inactivation in LSCC. SSTR5-AS1 may play antitumor role in LSCC and may be regulated by the hypermethylation of the same CpG sites with SSTR5. SSTR5-AS1 inhibits laryngeal carcinoma cells proliferation, migration, and invasion. SSTR5-AS1 increases the enrichment of MLL3 and H3K4me3 at the promoter region of SSTR5 by interacting with MLL3 and further induces the transcription of SSTR5. Furthermore, SSTR5-AS1 interacts with and recruits TET1 to its target gene E-cadherin to activate its expression.

**Conclusion:**

These findings suggest that the identified lncRNAs and mRNAs may be potential biomarkers in metastatic LSCC, and SSTR5-AS1 may act as a tumor suppressor as well as a potential biomarker for antitumor therapy.

**Electronic supplementary material:**

The online version of this article (10.1186/s13072-019-0283-8) contains supplementary material, which is available to authorized users.

## Background

Head and neck squamous cell carcinoma (HNSC) is the sixth most common type of cancer worldwide [1]. Laryngeal cancer is one of the most common head and neck cancers with well-defined risk factors such as tobacco abuse. Laryngeal cancer is responsible for 13,430 new cases and 3620 cancer-related deaths annually in the USA [2]. The incidence and mortality of laryngeal cancer were 26,400 and 14,500 patients in China in the year of 2015 [3]. Laryngeal squamous cell carcinoma (LSCC) is the predominant pathological subtype of laryngeal cancer. Despite the improvements in diagnosis and treatment, there is no significant improvement in the 5-year survival rate for LSCC in the past 30 years (from 59.6 to 66.8%) [4]. The exact molecular mechanisms and prognostic factors of LSCC still remain unclarified. Thus, identifying specific biomarkers to enable effective targeted therapy strategies is urgent for the improvement in diagnosis, treatment, and prognosis of LSCC.

Screening and identification of the differentially expressed coding genes and noncoding RNAs may be crucial for the diagnosis, prognosis, and personalized treatment of LSCC. Long noncoding RNAs (lncRNAs) are generally greater than 200 bp in transcription size and identified as nonprotein-coding transcripts [5]. LncRNAs are implicated in diverse biological processes and disease-related pathways, such as immune response, epigenetic regulation, lineage commitment, alternative splicing, cell proliferation and differentiation, alternation of protein localization, modulation of protein activity, and precursors of small RNAs [6–8]. Although many efforts have been made to annotate lncRNAs, the functions of most lncRNAs are still unclarified and only a few dozen of lncRNAs have been well characterized such as HOTAIR [9], ANRIL [10], and MEG3 [11].

Accumulating evidences have identified the important roles of lncRNAs in the initiation and progression of various cancer types. Critical lncRNAs may serve as potential biomarkers for early diagnosis, prognosis, and potential therapy targets in certain carcinomas [12–14]. However, the global lncRNAs and mRNAs expression profiles for LSCC are limited. Only a few studies focused on the role of lncRNAs on LSCC occurrence and progression [15–17]. Because the databases of lncRNAs have been constantly updated and due to the strong heterogeneity of tumors, it is necessary for the intensive study of the contribution of lncRNAs in metastatic LSCC. In the present study, we screened expression profiles of mRNAs and lncRNAs in four advanced LSCC patients (with lymph node metastasis) with paired tumor tissues and corresponding normal tissues by microarrays and identified numerous differentially expressed transcripts. After the necessary verification of the transcripts expression in expanded samples, we experimentally validated the expression patterns of the remarkable low expressed gene, SSTR5, and its antisense lncRNA, SSTR5-AS1. We further analyzed the epigenetic inactivation mechanisms including methylation and acetylation of SSTR5 and SSTR5-AS1 and further examined the functional role of SSTR5-AS1 and its regulation on SSTR5 expression.

## Results

**Fig. 1:**
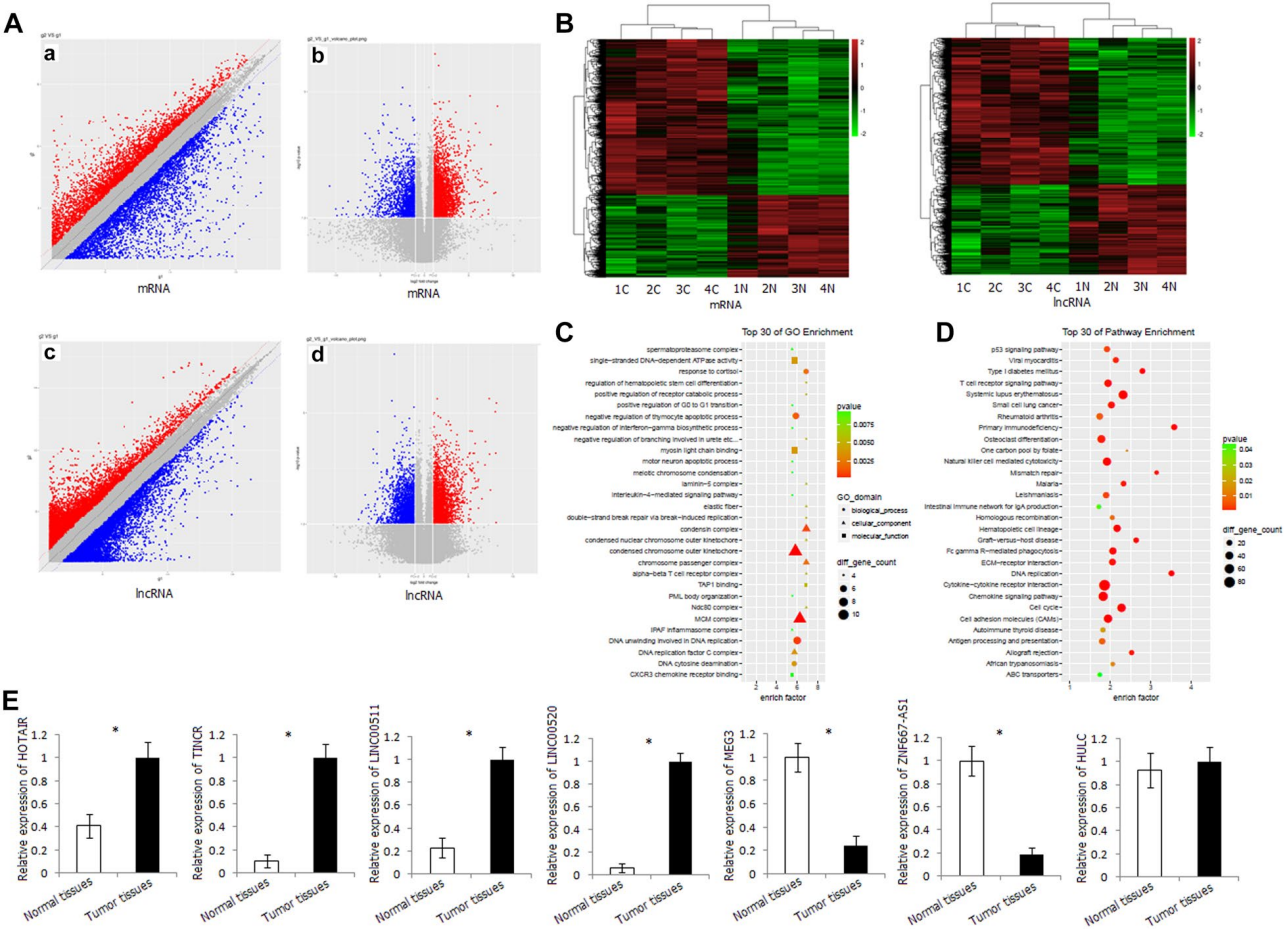
Differentially expressed mRNA and lncRNA in metastatic LSCC by microarray array. **A** Scatter plot and volcano plot of the mRNA and lncRNA distribution in LSCC tissues and corresponding normal tissues. **a** Scatter plot of mRNA distribution; **b** volcano plot of mRNA distribution; **c** scatter plot of lncRNA distribution; **d** volcano plot of lncRNA distribution. **B** Heatmap of the mRNA and lncRNA expression in LSCC tissues and corresponding normal tissues. **C** GO analysis of differentially expressed mRNA. **D** KEGG pathway analysis of differentially expressed mRNA. **E** Relative expression level of HOTAIR, TINCR, LINC00511, LINC00520, MEG3, ZNF667-AS1, and HULC in LSCC tissues and corresponding normal tissues detected by qRT-PCR. *P < 0.05

The gene expression profiles of LSCC tissues and corresponding normal tissues were significantly different. There are 2809 differentially expressed mRNAs, 1791 of which are upregulated and 1018 downregulated in cancer tissues. There are 3073 differentially expressed lncRNAs, 1967 of which are upregulated and 1106 downregulated in cancer tissues (fold change ≥ 2, *P* < 0.05) (Fig. 1A). Hierarchical cluster analyses indicated that the expression patterns in LSCC tissues were significantly different from those in corresponding normal tissues (Fig. 1B).

Gene Ontology (GO) and pathway analyses were performed to understand the biological processes. GO analysis revealed that aberrantly expressed mRNAs were mainly involved in three cellular component domains, including condensed chromosome outer kinetochore, MCM complex, and condensin complex; three biological process domains, including DNA unwinding involved in DNA replication, negative regulation of thymocyte apoptotic process, and response to cortisol; and two molecular function domains, including single-stranded DNA-dependent ATPase activity and myosin light chain binding (Fig. 1C). Pathway analysis revealed that aberrantly expressed mRNAs were mainly involved in immune-related signaling pathway and tumor-related pathway, including cytokine–cytokine receptor interaction, chemokine signaling pathway, natural killer cell-mediated cytotoxicity, T cell receptor signaling pathway, cell adhesion molecules, cell cycle, p53 signaling pathway, ECM–receptor interaction (Fig. 1D).

In order to confirm the results of microarray, we investigated the expression levels of some lncRNAs in 48 pairs of LSCC tissues and corresponding normal tissues. These lncRNAs included HOTAIR, TINCR, LINC00511, and LINC00520 (upregulated in LSCC tissues in microarray), MEG3 and ZNF667-AS1 (downregulated in LSCC tissues in microarray), and HULC (with no significant expression difference between tumors and corresponding normal tissues in microarray). The expression pattern of these lncRNAs in the LSCC tissues and corresponding normal tissues was in good agreement with that in the microarray, indicating the credibility and reliability of the results of microarray (Fig. 1E).

**Fig. 2:**
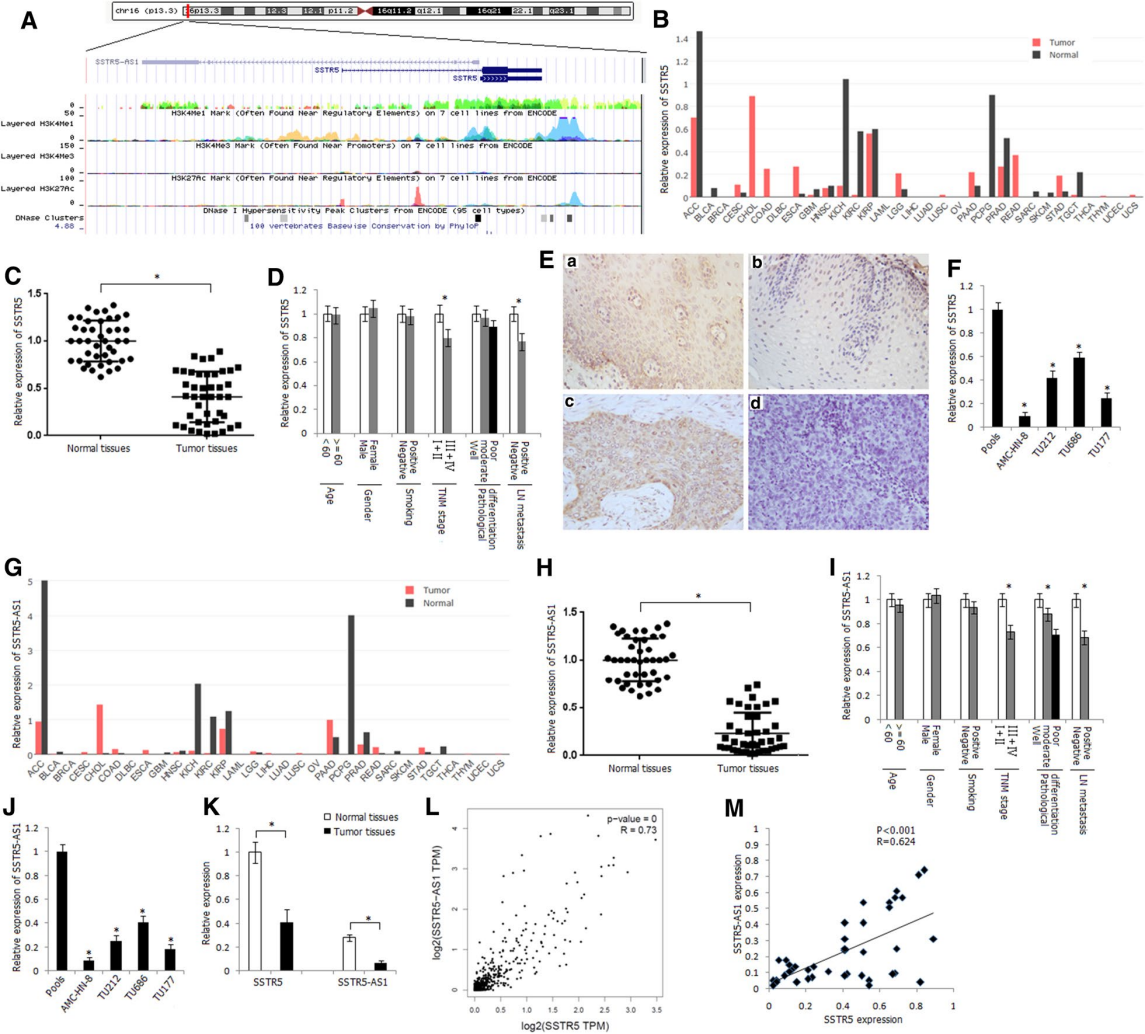
Expression status of SSTR5 and SSTR5-AS1 in LSCC tissues and laryngeal carcinoma cell lines. **A** Schematic representation of the genomic organization and epigenetic marks of SSTR5 and SSTR5-AS1 (data from UCSC Genome Browser). **B** Relative expression of SSTR5 in various tumor types in GEPIA dataset. *ACC* adrenocortical carcinoma, *BLCA* bladder urothelial carcinoma, *BRCA* breast invasive carcinoma, *CESC* cervical squamous cell carcinoma and endocervical adenocarcinoma, *CHOL* cholangio carcinoma, *COAD* colon adenocarcinoma, *DLBC* lymphoid neoplasm diffuse large B cell lymphoma, *ESCA* esophageal carcinoma, *GBM* glioblastoma multiforme, *HNSC* head and neck squamous cell carcinoma, *KICH* kidney chromophobe, *KIRC* kidney renal clear cell carcinoma, *KIRP* kidney renal papillary cell carcinoma, *LAML* acute myeloid leukemia, *LGG* brain lower grade glioma, *LIHC* liver hepatocellular carcinoma, *LUAD* lung adenocarcinoma, *LUSC* lung squamous cell carcinoma, *MESO* mesothelioma, *OV* ovarian serous cystadenocarcinoma, *PAAD* pancreatic adenocarcinoma, *PCPG* pheochromocytoma and paraganglioma, *PRAD* prostate adenocarcinoma, *READ* rectum adenocarcinoma, *SARC* sarcoma, *SKCM* skin cutaneous melanoma, *STAD* stomach adenocarcinoma, *TGCT* testicular germ cell tumors, *THCA* thyroid carcinoma, *THYM* thymoma, *UCEC* uterine corpus endometrial carcinoma, *UCS* uterine carcinosarcoma, *UVM* uveal melanoma. **C** Relative expression level of SSTR5 in LSCC tissues and corresponding normal tissues, as determined by qRT-PCR method. *P < 0.05. **D** Relative expression level of SSTR5 in different subgroups. *P < 0.05. **E** Immunohistochemical staining of SSTR5 in LSCC tumor tissues and normal tissues (SP × 400). **a** Positive staining of SSTR5 in normal tissue; **b** negative staining of SSTR5 in normal tissue; **c** positive staining of SSTR5 in LSCC tissue; **d** negative staining of SSTR5 in LSCC tissue. **F** Relative expression level of SSTR5 in human laryngeal carcinoma cell lines. Pools: average expression in ten normal tissues was used as normal control. *Compared with pools, *P* < 0.05. **G** Relative expression level of SSTR5-AS1 in various tumor types in GEPIA dataset. **H** Relative expression level of SSTR5-AS1 in LSCC tissues and corresponding normal tissues, as determined by qRT-PCR method. *P < 0.05. I. Relative expression level of SSTR5-AS1 in different subgroups. *P < 0.05. **J** Relative expression level of SSTR5-AS1 in human laryngeal carcinoma cell lines. *Compared with pools, *P* < 0.05. **K** Expression comparison of SSTR5 and SSTR5-AS1 in LSCC tissues and corresponding normal tissues. *P < 0.05. **L** Correlation between the expression of SSTR5 and SSTR5-AS1 in HNSC tissues in GEPIA dataset. **M** Correlation between the expression of SSTR5 and SSTR5-AS1 in LSCC tissues

We focused our attention on a number of lncRNAs which with relatively high-fold-change expression and target gene prediction suggested associations with important biological function in cis and in trans. Among the differentially expressed lncRNAs, there are 185 differentially expressed antisense lncRNA transcripts. For these antisense lncRNAs, we identified one transcript with 0.03-fold change downregulation in the tumors and its sense transcript SSTR5 with 0.06-fold change downregulation in tumor tissues (Additional file 1: Table S5). The computational analysis of coding potential suggested a very low coding potential of SSTR5-AS1 gene. Schematic representation of the genomic organization of SSTR5 and SSTR5-AS1 is shown in Fig. 2A. There are two transcripts of SSTR5, and the main transcript (NM_001053) is located at chr16: 1078781-1081454 (GRCh38/hg38). SSTR5-AS1 (NR_027242) is located at chr16: 1064081-1078731 (GRCh38/hg38), and there are 51 bases interval between the transcriptional start site of SSTR5 and SSTR5-AS1.

We first scanned relative expression level of SSTR5 in various tumor types in GEPIA dataset and found significant downregulation of SSTR5 in most of the tumor types including adrenocortical carcinoma (ACC), kidney renal clear cell carcinoma (KIRC), and pheochromocytoma and paraganglioma (PCPG) (Fig. 2B). The mRNA expression level of SSTR5 in LSCC tumor tissues was significantly decreased than that in corresponding normal tissues in the present study (*P* < 0.01) (Fig. 2C). The expression level of SSTR5 in LSCC tumor tissues was associated with TNM stage and lymph node metastasis (*P* < 0.05) (Fig. 2D). The pattern of immunohistochemical staining of SSTR5 was cytoplasmic (Fig. 2E). The positive protein expression frequency of SSTR5 in tumor tissues (33.3%, 16/48) was significantly lower than that in corresponding normal tissues (72.9%, 35/48) (*P* < 0.05). Protein expression of SSTR5 was associated with TNM stage and lymph node metastasis (*P* < 0.05) (Table 1). We further detected the expression level of SSTR5 in laryngeal carcinoma cell lines and found significant downregulation of SSTR5 in four laryngeal carcinoma cells (Fig. 2F).

**Table 1:** Protein expression and methylation status of SSTR5 in LSCC tumor tissues

Groups	*N*	Protein expression		Methylation frequency (promoter)		Methylation frequency (Exon 1)	
		*n* (%)	*P*	*n* (%)	*P*	*n* (%)	*P*
Age							
< 60	25	9 (36.0)		4 (16.0)		13 (52.0)	
≥ 60	23	7 (30.4)	0.683	5 (21.7)	0.611	14 (60.9)	0.536
Gender							
Male	46	15 (32.6)		9 (19.6)		26 (56.5)	
Female	2	1 (50.0)	0.610	0 (0.0)	0.488	1 (50.0)	0.856
Smoking							
Negative	10	4 (40.0)		2 (20.0)		5 (50.0)	
Positive	38	12 (31.6)	0.615	7 (18.4)	0.909	22 (57.9)	0.654
TNM stage							
I + II	19	10 (52.6)		3 (15.8)		7 (36.8)	
III + IV	29	6 (20.7)	0.022	6 (20.7)	0.671	20 (68.9)	0.028
Pathological differentiation of tumor							
Well	20	9 (45.0)		3 (15.0)		8 (40.0)	
Moderate	16	5 (31.3)		3 (18.7)		10 (62.5)	
Poor	12	2 (16.7)	0.252	3 (25.0)	0.782	9 (75.0)	0.128
LN metastasis							
Negative (N0)	22	11 (50.0)		4 (18.2)		8 (36.4)	
Positive (N1/2/3)	26	5 (19.2)	0.024	5 (19.2)	0.926	19 (73.1)	0.011

Significant downregulation of SSTR5-AS1 was also found in various tumor types in GEPIA dataset (Fig. 2G). The expression level of SSTR5-AS1 was significantly decreased in LSCC tissues (Fig. 2H) and was correlated with TNM stage, pathological differentiation, and lymph node metastasis in the present study (Fig. 2I). Downregulation of SSTR5-AS1 was also detected in the four laryngeal carcinoma cells (Fig. 2J). Higher general mRNA expression level of SSTR5 than SSTR5-AS1 was found either in LSCC tissues or in corresponding normal tissues (Fig. 2K). However, the average fold change of SSTR5-AS1 expression level was higher than SSTR5 expression level in LSCC tissues compared with corresponding normal tissues (Fig. 2K). A positive correlation was shown between the expression levels of SSTR5 and SSTR5-AS1 in HNSC tissues in GEPIA dataset (Fig. 2L). In the present study, we also detected positive correlation between the expression levels of SSTR5 and SSTR5-AS1 in LSCC tissues (Fig. 2M).

**Fig. 3:**
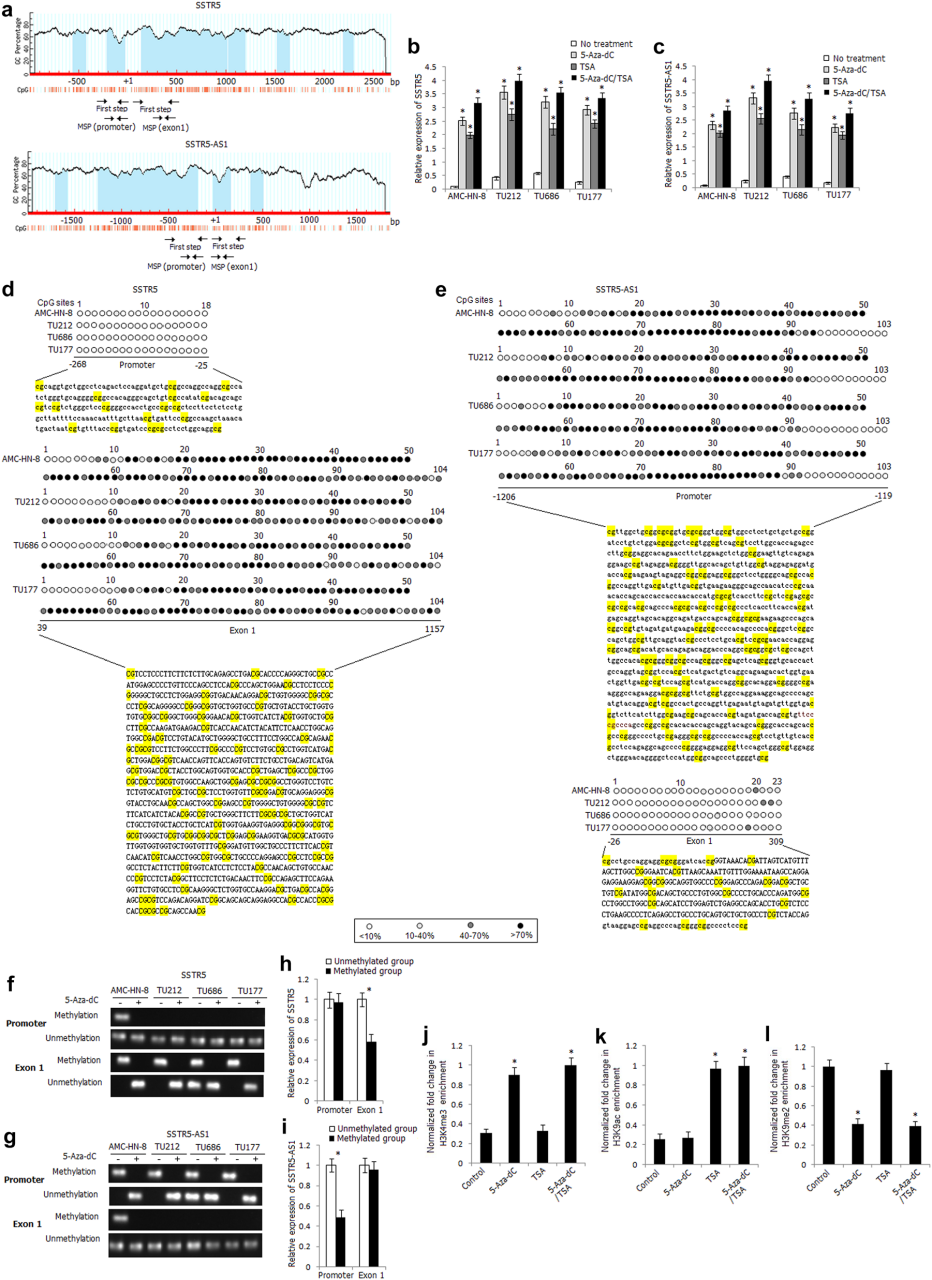
Epigenetic regulation mechanisms of inactivation of SSTR5 and SSTR5-AS1 in LSCC. **a** Schematic structure of SSTR5 and SSTR5-AS1 CpG islands predicted by MethPrimer. MSP regions analyzed are indicated in the different regions. Relative expression levels of SSTR5 (**b**) and SSTR5-AS1 (**c**) in 5-Aza-dC-, TSA-, 5-Aza-dC/TSA-treated laryngeal carcinoma cells. *Compared with untreated cells, *P* < 0.05. D, E. High-resolution mapping of the methylation status of every CpG site in the promoter and exon 1 regions of SSTR5 (**d**) and SSTR5-AS1 (**e**) by BGS assay in four laryngeal carcinoma cells. Each CpG site is shown at the top row as an individual number. Percentage methylation was determined as percentage of methylated cytosines from eight to ten sequenced colonies. The color of circles for each CpG site represents the percentage of methylation. Methylation status of the promoter and exon 1 regions of SSTR5 (**f**) and SSTR5-AS1 (**g**) determined by BS-MSP analysis in laryngeal carcinoma cells treated or untreated with 5-Aza-dC. Relative expression levels of SSTR5 (**h**) and SSTR5-AS1 (**i**) in the tumor tissues with and without methylation of the promoter and exon 1 regions. *P < 0.05. ChIP assay was used to determine the enrichment of H3K4me3 (**j**), H3K9ac (**k**), H3K9me2 (**l**) within the SSTR5 promoter in AMC-HN-8 cells. *Compared with untreated cells, *P* < 0.05

To explore the inactivation mechanisms of SSTR5 and SSTR5-AS1, we analyzed the distribution of CpG islands of both genes and found obvious CpG islands in the promoter and exon 1 regions of SSTR5 and SSTR5-AS1 (Fig. 3a). Because SSTR5 and SSTR5-AS1 are head-to-head genes which are amplified from opposite direction, the distribution of CpG sites is identical, just reverse distribution. Furthermore, bioinformatics analysis found abundant H3K4me3 signals in the promoter region of SSTR5 and SSTR5-AS1, suggesting that SSTR5 and SSTR5-AS1 might be regulated by histone modification. We treated the laryngeal carcinoma cells with DNA methyltransferase inhibitor 5-Aza-dC and/or histone deacetylase inhibitor TSA. As shown in Fig. 3b, c, the expression levels of SSTR5 and SSTR5-AS1 were significantly increased in the 5-Aza-dC-, TSA-, 5-Aza-dC/TSA-treated laryngeal carcinoma cells, and the effect was more apparent in the 5-Aza-dC/TSA-treated cells, indicating that the expression of SSTR5 and SSTR5-AS1 might be co-regulated by DNA methylation and histone modification.

We detected the methylation status of CpG sites in the promoter and exon 1 regions of SSTR5 and SSTR5-AS1 in four laryngeal carcinoma cells by BGS assay. As shown in Fig. 3d, frequent CpG sites methylation was observed in the exon 1 region of SSTR5, while frequent methylated CpG sites were located in the promoter region of SSTR5-AS1 (Fig. 3e), which were corresponding to the CpG sites in the exon 1 of SSTR5. The two pairs of BS-MSP primers were designed according to the distribution of methylated CpG sites in the promoter and exon 1 regions of SSTR5 and SSTR5-AS1. As shown in Fig. 3f, g, apparent methylation of exon 1 of SSTR5 and promoter of SSTR5-AS1 was detected in laryngeal carcinoma cells before 5-Aza-dC treatment and the methylation status was completely reversed after 5-Aza-dC treatment, suggesting the important role of exon 1 methylation of SSTR5 and promoter methylation of SSTR5-AS1 in gene silencing. The methylation status of the promoter and exon 1 regions of SSTR5 and SSTR5-AS1 was further successfully detected in all tissue specimens by BS-MSP method. For SSTR5, of primary LSCC tissues and corresponding normal tissues, hypermethylation was observed in 18.7% (9/48) and 10.4% (5/48) at promoter region, 56.3% (27/48) and 12.5% (6/48) at exon 1 region, respectively (Additional file 2: Table S6). The methylation frequency of exon 1 region of SSTR5 in LSCC tissues was significantly higher than that in corresponding normal tissues (*P* < 0.05). The methylation status of promoter region of SSTR5 in LSCC tissues was not associated with any clinicopathologic characteristics, while the methylation status of exon 1 in LSCC tissues was associated with TNM stage and lymph node metastasis (*P* < 0.05) (Table 1). For SSTR5-AS1, of primary LSCC tissues and corresponding normal tissues, hypermethylation was observed in 52.1% (25/48) and 14.6% (7/48) at promoter region, 16.7% (8/48) and 12.5% (6/48) at exon 1 region, respectively (Additional file 3: Table S7). The methylation frequency of promoter region of SSTR5-AS1 in LSCC tissues was significantly higher than that in corresponding normal tissues (*P* < 0.05). The methylation status of promoter region of SSTR5-AS1 in LSCC tissues was associated with TNM stage, pathological differentiation, and lymph node metastasis (*P* < 0.05), while the methylation status of exon 1 of SSTR5-AS1 in LSCC tissues was not associated with any clinicopathologic characteristics (Table 2).

**Table 2:** Methylation status of SSTR5-AS1 in LSCC tumor tissues

Groups	*N*	Methylation frequency			
		Promoter		Exon 1	
		*n* (%)	*P*	*n* (%)	*P*
Age					
< 60	25	12(48.0)		4(16.0)	
≥ 60	23	13(56.5)	0.555	4(17.4)	0.897
Gender					
Male	46	24(52.2)		8(17.4)	
Female	2	1(50.0)	0.952	0(0.0)	0.518
Smoking					
Negative	10	4(40.0)		2(20.0)	
Positive	38	21(55.3)	0.390	6(15.8)	0.751
TNM stage					
I + II	19	6(31.6)		3(15.8)	
III + IV	29	19(65.5)	0.021	5(17.2)	0.895
Pathological differentiation of tumor					
Well	20	6(30.0)		2(10.0)	
Moderate	16	10(62.5)		3(18.7)	
Poor	12	9(75.0)	0.028	3(25.0)	0.525
LN metastasis					
Negative (N0)	22	7(31.8)		3(13.6)	
Positive (N1/2/3)	26	18(69.2)	0.010	5(19.2)	0.604

The mRNA expression level of SSTR5 in LSCC tissues with hypermethylation of exon 1 was significantly decreased than that with unmethylation of this region (*P* < 0.05); however, the expression level of SSTR5 was not associated with methylation status of promoter region (*P* > 0.05) (Fig. 3h). The protein expression of SSTR5 was also significantly correlated with exon 1 methylation status and was not correlated with promoter methylation status (Additional file 4: Table S8). As shown in Fig. 3i, the expression level of SSTR5-AS1 in LSCC tissues with hypermethylation of promoter region was significantly lower than that with unmethylation of this region (*P* < 0.05).

To determine the potential role of histone modifications on SSTR5 downregulation, the presence of active (H3K4me3, H3K9ac) and inactive (H3K9me2) histone modifications at SSTR5 promoter was further examined by chromatin immunoprecipitation assay in AMC-HN-8 cells (Fig. 3j–l). The repressive mark H3K9me2 was most enriched in AMC-HN-8 cells than active mark H3K4me3 and H3K9ac. Increased enrichment of H3K4me3 and decreased enrichment of H3K9me2 were detected in 5-Aza-dC-treated AMC-HN-8 cells, and significant increased enrichment of H3K9ac was detected in TSA-treated AMC-HN-8 cells, indicating that in addition to DNA methylation, histone modification is also involved in the regulation of SSTR5 expression.

**Fig. 4:**
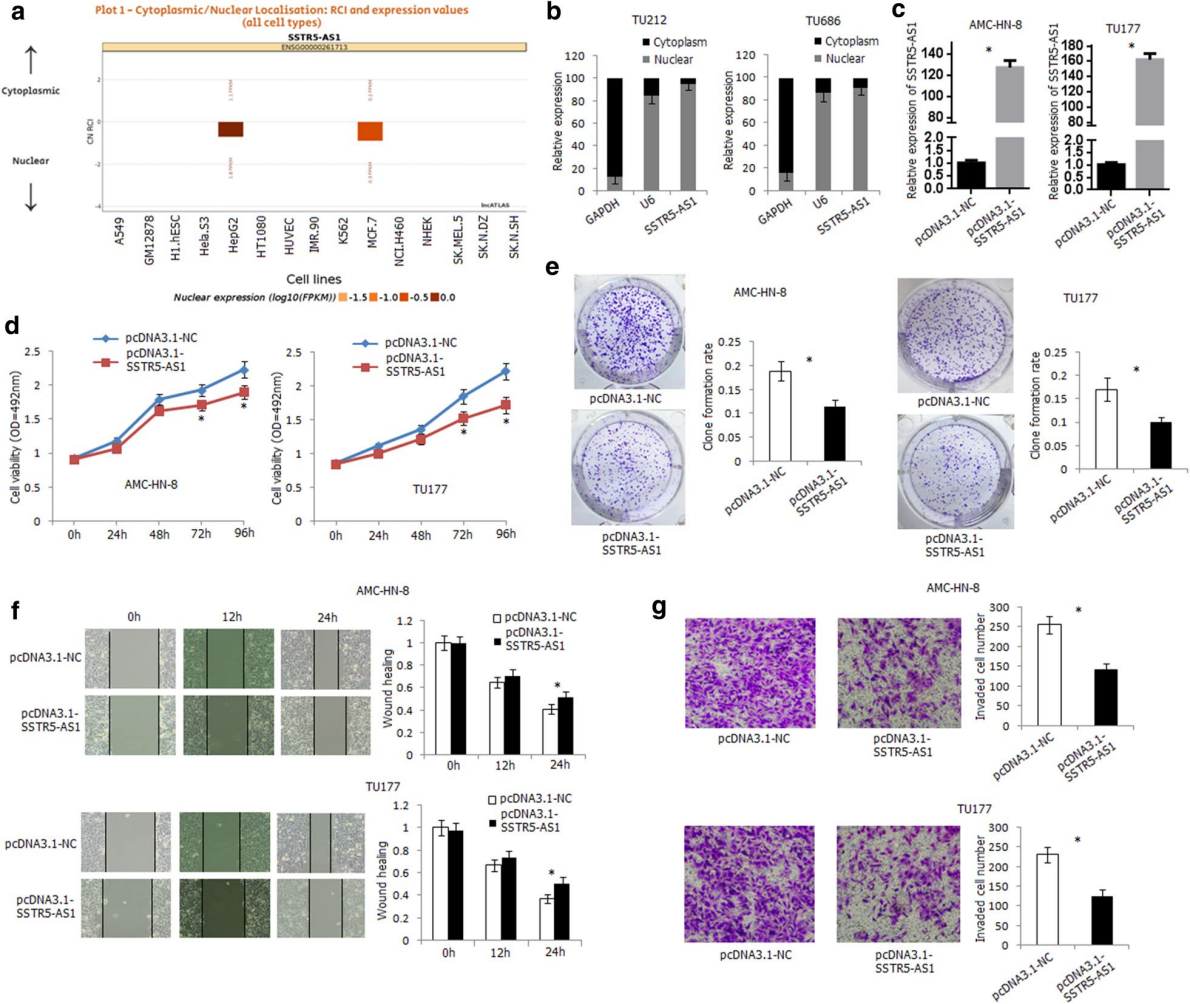
Functional analysis of SSTR5-AS1 in human laryngeal carcinoma cells. **a** Subcellular localization of SSTR5-AS1 in various cells predicted by lncATLAS. **b** Expression levels of SSTR5-AS1 in nuclear and cytoplasmic fractions of TU212 and TU686 cells detected by qRT-PCR method. GAPDH: cytoplasmic control; U6: nuclear control. **c** Significant upregulation of SSTR5-AS1 was detected by qRT-PCR in SSTR5-AS1-transfected AMC-HN-8 and TU177 cells. *Compared with empty vector transfected cells, *P* < 0.05. **d** Over-expression of SSTR5-AS1 inhibited AMC-HN-8 and TU177 cells proliferation. *Compared with empty vector, *P* < 0.05. **e** Over-expression of SSTR5-AS1 inhibited AMC-HN-8 and TU177 cells colony formation ability. *Compared with empty vector, *P* < 0.05. **f** Over-expression of SSTR5-AS1 inhibited AMC-HN-8 and TU177 cells migration ability. *Compared with empty vector, *P* < 0.05. **g** Over-expression of SSTR5-AS1 inhibited AMC-HN-8 and TU177 cells invasion ability. *Compared with empty vector, *P* < 0.05

The function of SSTR-AS1 was further investigated in laryngeal carcinoma cell lines. We first scanned and detected the subcellular location of SSTR5-AS1 in cells. SSTR5-AS1 was mainly located in the nucleus of HepG2 and MCF7 in lncATLAS (http://lncatlas.crg.eu) (Fig. 4a). In the present study, SSTR5-AS1 was distributed mainly in the nucleus of TU212 and TU686 cells (Fig. 4b). The construct containing SSTR5-AS1 transcript (pcDNA3.1-SSTR5-AS1) was transfected into AMC-HN-8 and TU177 cells, and the expression level of SSTR5-AS1 was significantly increased in the transfected cells (Fig. 4c). Overexpression of SSTR5-AS1 led to a significant inhibition of AMC-HN-8 and TU177 cells proliferation by CCK-8 and clone formation assay (Fig. 4d, e). Wound healing assay demonstrated that overexpression of SSTR5-AS1 inhibited the migration ability of AMC-HN-8 and TU177 cells (Fig. 4f). Furthermore, overexpression of SSTR5-AS1 inhibited the invasion ability of AMC-HN-8 and TU177 cells by transwell invasion assay (Fig. 4g). For the nuclear localization of SSTR5-AS1, we further knocked down SSTR5-AS1 using ASO in TU686 cells (Additional file 5: Fig. S1A). Transfection of ASO-SSTR5-AS1 in TU686 cells led to increased proliferation, migration, and invasion ability of the cells (Additional file 5: Fig. S1B, C, D).

**Fig. 5:**
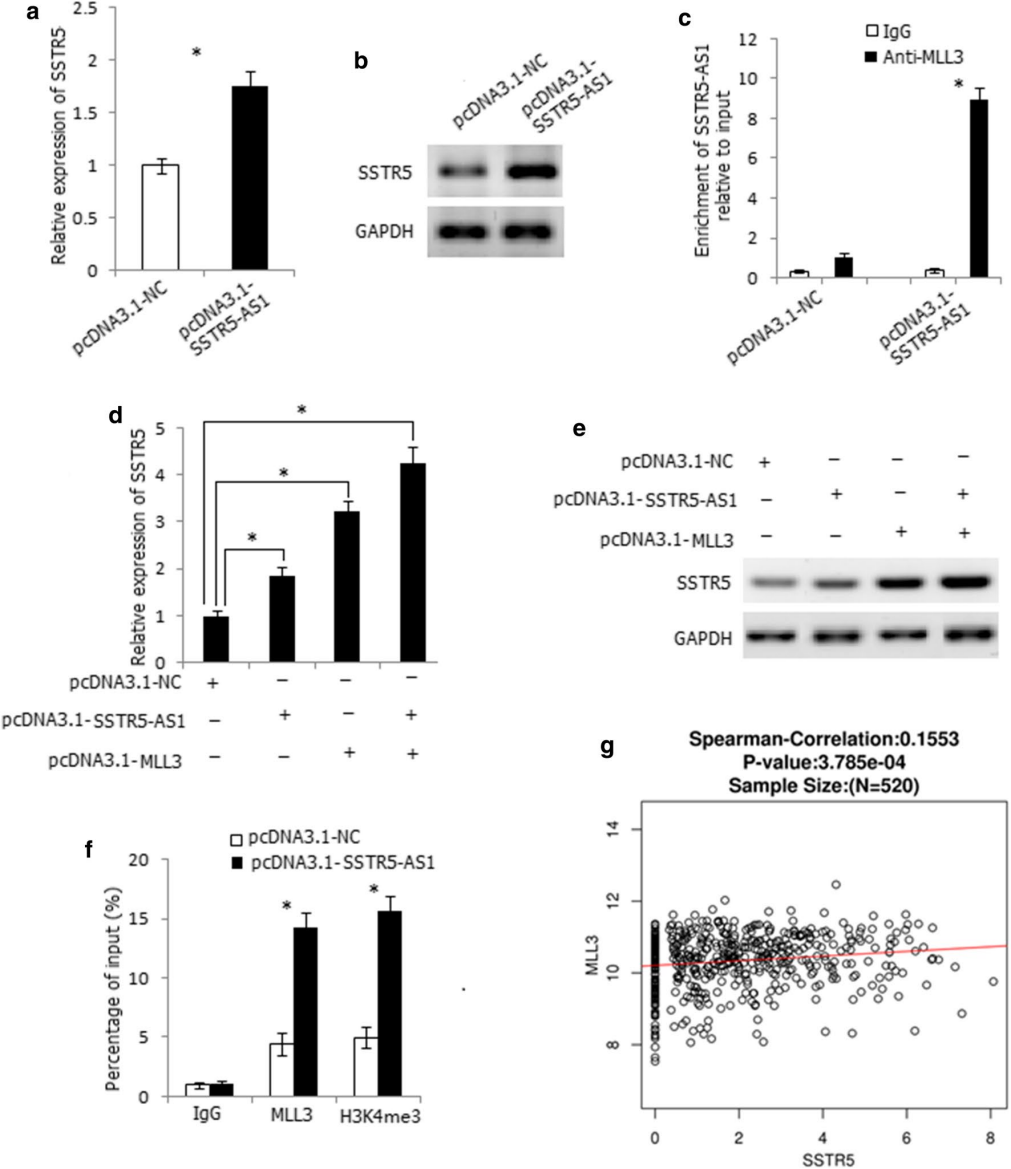
SSTR5-AS1 upregulates SSTR5 expression through interacting with MLL3. **a** Overexpression of SSTR5-AS1 increased mRNA expression level of SSTR5 in AMC-HN-8 cells. *P < 0.05. **b** Overexpression of SSTR5-AS1 increased protein expression level of SSTR5 in AMC-HN-8 cells. **c** RIP assay showed the binding of SSTR5-AS1 to MLL3 in SSTR5-AS1-transfected AMC-HN-8 cells. *P < 0.05. **d** Co-expression of SSTR5-AS1 and MLL3 had a synergistic promoting effect on the mRNA up regulation of SSTR5. *P < 0.05. **e** Co-expression of SSTR5-AS1 and MLL3 had a synergistic promoting effect on the protein overexpression of SSTR5. **f** ChIP assay revealed that overexpression of SSTR5-AS1 increased the enrichment of MLL3 and the level of H3K4me3 at the promoter region of SSTR5. *Compared with empty vector, *P* < 0.05. **g** The expression level of SSTR5 was positively correlated with MLL3 in HNSC in LinkedOmics dataset

Antisense lncRNAs have been reported to regulate the expression and function of sense mRNA in cis [18, 19]. This prompts us to analyze whether SSTR5-AS1 regulates the expression of SSTR5. Overexpression of SSTR5-AS1 increased the mRNA and protein expression levels of SSTR5 in AMC-HN-8 cells (Fig. 5a, b). SSTR5-AS1 was mainly distributed in the nucleus of cells, so we considered if SSTR5-AS1 regulated the expression of SSTR5 in nucleus by binding some proteins which play roles in the nucleus, such as transcription factors and epigenetic regulatory enzymes. We noticed abundant H3K4me3 signals in the promoter region of SSTR5, suggesting that SSTR5 might be regulated by histone methylation. Myeloid/lymphoid or mixed lineage leukemia (MLL) family genes are the mostly important enzymes that modify H3K4 methylation [20, 21], so we wondered whether MLL family genes might participate in SSTR5 regulation by binding to SSTR5-AS1. We predicted the binding ability of MLL1 to MLL5 with SSTR5-AS1 by RPISeq (http://pridb.gdcb.iastate.edu/RPISeq/) and found the greatest interaction probability between MLL3 protein and SSTR5-AS1 (RF: 0.65 and SVM: 0.66). RNA immunoprecipitation assay demonstrated the binding of SSTR5-AS1 to MLL3 protein in SSTR5-AS1-transfected AMC-HN-8 cells (Fig. 5c). Overexpression of MLL3 increased the mRNA and protein expression level of SSTR5; moreover, co-overexpression of MLL3 and SSTR5-AS1 demonstrated stronger increasing effect of SSTR5 expression level both in transcriptional and translational levels, indicating the synergetic effect of MLL3 and SSTR5-AS1 on SSTR5 regulation (Fig. 5d, e). ChIP assay further revealed that overexpression of SSTR5-AS1 increased the enrichment of MLL3 and the level of H3K4me3 at the promoter region of SSTR5 (Fig. 5f). Moreover, SSTR5 was positively correlated with MLL3 expression in HNSC by LinkedOmics (Fig. 5g). Thus, SSTR5-AS1 increased the enrichment of MLL3 and H3K4me3 at the promoter region of SSTR5 by interacting with MLL3 and further induced the transcription of SSTR5.

**Fig. 6:**
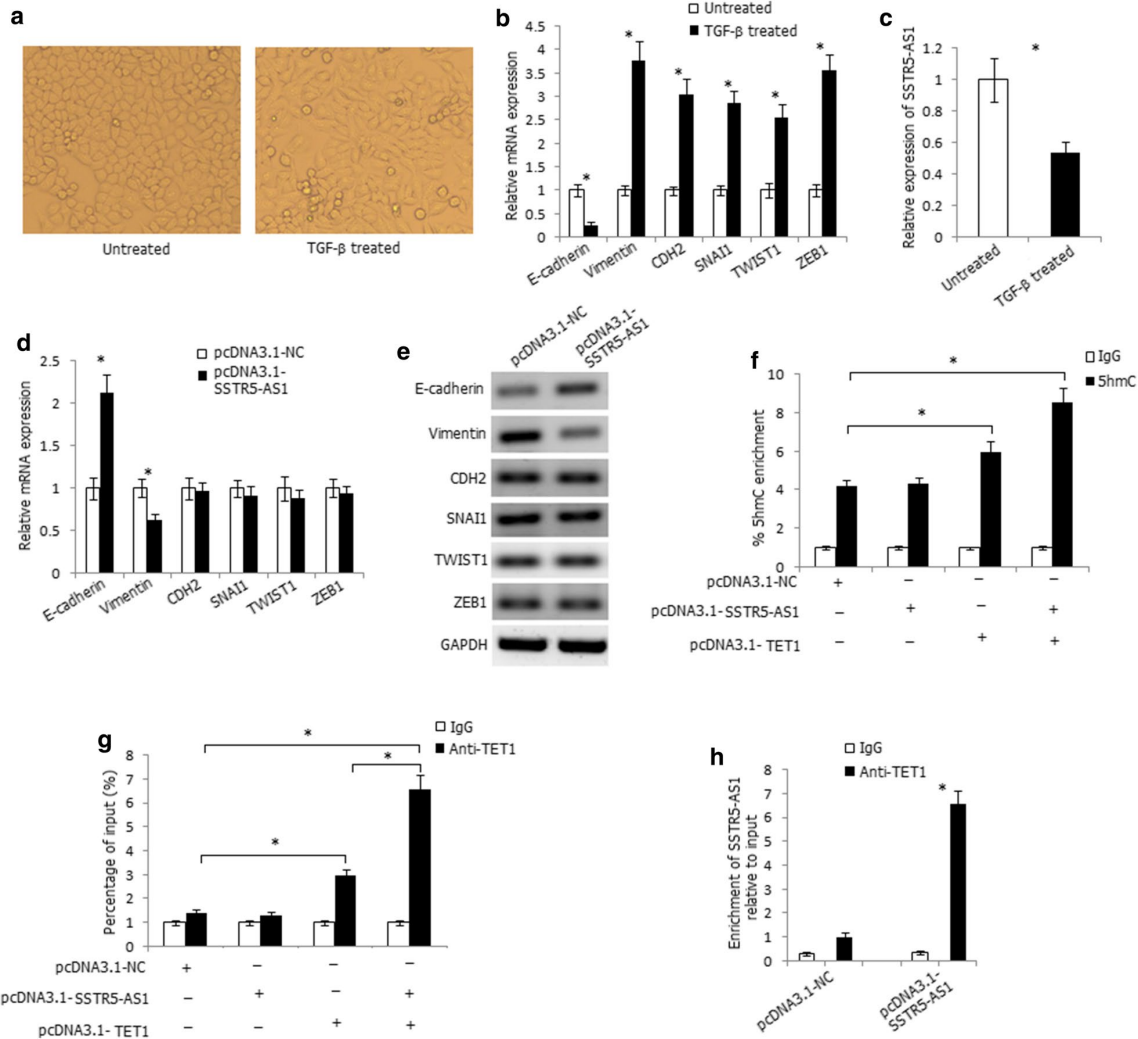
SSTR5-AS1 recruits TET1 to its target gene E-cadherin to increase its expression through increasing 5hmC levels at the promoter region of E-cadherin. **a** Phase-contrast micrographs of AMC-HN-8 cells treated with TGF-β1 for 21d. **b** Relative mRNA expression levels of EMT markers in AMC-HN-8 cells treated with TGF-β1. *Compared with untreated cells, *P* < 0.05. **c** Relative expression level of SSTR5-AS1 in AMC-HN-8 cells treated with TGF-β1. *Compared with untreated cells, *P* < 0.05. **d** Relative mRNA expression levels of EMT markers in SSTR5-AS1-transfected AMC-HN-8 cells. *P < 0.05. **e** Relative protein expression levels of EMT markers in SSTR5-AS1-transfected AMC-HN-8 cells. **f** The 5hmC enrichment at the promoter region of E-cadherin was determined by hMeDIP-qPCR method. *P < 0.05. **g** Overexpression of SSTR5-AS1 increased the enrichment of TET1 at the promoter region of E-cadherin detected by ChIP assay. *P < 0.05. **h** RIP assay showed the binding of SSTR5-AS1 to TET1 in SSTR5-AS1-transfected AMC-HN-8 cells. *P < 0.05

We further investigated the role of SSTR5-AS1 in EMT due to the inhibitory effect of SSTR5-AS1 on laryngeal carcinoma cells migration and invasion. We treated AMC-HN-8 cells with TGF-β for 21 days, which caused the cells to undergo EMT, as indicated by a spindle-shaped appearance (Fig. 6a) and accompanied with decreased expression of the epithelial marker E-cadherin and increased expression of the mesenchymal markers and transcription factors vimentin, CDH2, SNAI1, TWIST1, and ZEB1 (Fig. 6b). The expression level of SSTR5-AS1 was significantly decreased in the TGF-β-treated cells (Fig. 6c). Overexpression of SSTR5-AS1 increased the mRNA and protein expression levels of E-cadherin, decreased the mRNA and protein expression levels of vimentin, and, however, had no effect on the expression levels of CDH2, SNAI1, TWIST1, and ZEB1 (Fig. 6d, e), suggesting that SSTR5-AS1 may inhibit EMT process through regulating the expression of E-cadherin and Vimentin in LSCC.

For the more evident expression alterations of E-cadherin in SSTR5-AS1-transfected cells, we further focused on the regulatory effect of SSTR5-AS1 on E-cadherin. Because SSTR5-AS1 was mainly distributed in the nucleus of cells, we considered the regulatory role of SSTR5-AS1 at the genomic level. Promoter CpG sites hypermethylation of E-cadherin is a recognized mechanism of its inactivation in numerous cancers. Recently, the ten–eleven translocation (TET) gene family (including TET1, TET2, and TET3) has been proved to hydrolyze 5′-methylcytosine (5′-mc) to 5′-hydroxymethylcytosine (5′-hmc) and finally erase the methyl group from the CpG dinucleotides to activate gene expression. E-cadherin is the reported TET target gene [22, 23], so we considered whether SSTR5-AS1 interacts with TET to regulate the expression of E-cadherin. We predicted the binding ability of TET1, TET2, and TET3 to SSTR5-AS1 by RPISeq and found the greatest interaction probability between TET1 protein and SSTR5-AS1 (RF: 0.75 and SVM: 0.70). The hMeDIP-qPCR assay was used to track the 5hmC change in the CpG-rich regions of E-cadherin promoters, and co-expression of SSTR5-AS1 and TET1 in AMC-HN-8 cells significantly increased 5hmC levels at the promoter regions of E-cadherin (Fig. 6f). ChIP-qPCR analysis further demonstrated the binding of TET1 to the promoter regions of E-cadherin, and overexpression of SSTR5-AS1 significantly increased the enrichment of TET1 at the promoter regions of E-cadherin (Fig. 6g). Notably, RNA immunoprecipitation assay demonstrated the binding of SSTR5-AS1 to TET1 protein in SSTR5-AS1-transfected AMC-HN-8 cells (Fig. 6h). Thus, SSTR5-AS1 interacts with and recruits TET1 to its target gene E-cadherin to hydrolyze 5′-mc to 5′-hmc and further activates E-cadherin expression.

## Discussion

As an important member of ncRNAs, lncRNAs may play essential regulatory role in driving cancer [24]. A large number of lncRNAs are dynamically expressed across different developmental stages and demonstrate tissue-specific and differentiation-specific expression patterns [25]. With the development of microarrays and high-throughput RNA sequencing (RNA-seq), it is possible to examine the transcriptomes to an unprecedented degree, and a huge amount of lncRNAs is found to be uniquely expressed in specific tumor types [26]. However, the contribution of lncRNAs in metastatic LSCC remains largely unclarified. In the present study, we investigated the expression differences of mRNAs and lncRNAs between the metastatic LSCC tissues and corresponding normal tissues using microarray and identified amount of differentially expressed mRNAs and lncRNAs, and the majority of the lncRNAs have not yet been reported.

There are several differentially expressed lncRNAs with fold change greater than ± 10, which include intergenic lncRNAs, intronic and exonic antisense lncRNAs, bidirectional lncRNAs, and microRNA host lncRNAs. Of which, AC098973.2 demonstrated the greatest fold change (> 200); however, there is no research report about the roles of it in LSCC. Among the differentially expressed lncRNAs with greater fold change, CCAT1 (fold change 35) has recently validated the oncogenic roles in several cancer types [27, 28]. MEG3 is another lncRNA with fold change 0.09 in the present study, which has been widely studied in carcinomas and may be involved in tumorigenesis as tumor suppressor [29, 30]. In order to confirm the results of microarray, we validated the expression of seven lncRNAs which have been reported in other cancers. The validation results were highly in great agreement with those in microarray, and the expression tendency of HOTAIR, TINCR, LINC00511, LINC00520, MEG3, and ZNF667-AS1 was also consistent with literature reports in other carcinomas. However, HULC, which played oncogenic roles in several cancers, including liver cancer and prostate cancer [31, 32], demonstrated no significant expression difference between LSCC tumors and corresponding normal tissues in the present study, indicating that the expression pattern of lncRNAs may have cell-type specificity, and further studies should be addressed to detect the roles of tumor-specific lncRNAs.

Approximately 50–70% of lncRNAs are classified as antisense lncRNAs [33]. The importance of antisense lncRNAs has been overlooked for many years due to their low expression level and heterogeneity; however, antisense lncRNAs have attracted increased attention due to their locus-specific effects in recent years. Studies have shown that antisense lncRNAs may play important roles in a highly cell-type-specific manner, exerting cis- or trans-effects on protein-coding genes [34]. For example, KRT7-AS is activated in gastric cancers and supports cancer cell progression by increasing KRT7 expression [35]. AChE-AS represses AChE expression via epigenetic modification of the AChE promoter region and demonstrates an anti-apoptotic effect in hepatocellular carcinoma cells [36]. In the current study, we detected 185 differentially expressed antisense lncRNA transcripts by microarray and noticed that both SSTR5-AS1 and its sense protein-coding genes SSTR5 were with greater fold change. Moreover, by genomic sequence analysis, obvious CpG islands were found in the promoter and exon 1 regions of SSTR5 and SSTR5-AS1, indicating the possible epigenetic regulation mechanisms on their expression regulation. Somatostatin (SMS), a neuropeptide with multiple physiological activities, has recently been shown to exert anti-tumor effect by affecting tumor cell proliferation, angiogenesis, apoptosis, and the host’s immune response [37]. Cellular functions of SMS are induced by binding to G-protein-coupled plasma membrane receptors SSTR1, 2A, 2B, 3, 4, and 5 on normal and tumoral tissue targets. It has been well characterized that various carcinomas express SSTRs with variable abundance of subtypes [38]. In the present study, we verified the tumor suppressor role of SSTR5 and SSTR5-AS1 in LSCC progression; DNA hypermethylation and histone modification may co-regulate the expression of SSTR5 and SSTR5-AS1. Furthermore, SSTR5-AS1 may exert its anti-tumor effect by inhibiting laryngeal carcinoma cells proliferation, migration, and invasion.

A large amount of antisense lncRNAs can regulate the expression of sense protein-coding genes in cis, and lncRNAs located in the nucleus can guide and recruit DNA or histone protein modification enzymes, or transcription factors to specific genomic loci to regulate gene expression [39]. In the present study, we detected that SSTR5-AS1 could upregulate SSTR5 expression by interacting with MLL3. MLL3 is a member of the MLL family and encodes a nuclear protein with an AT hook DNA-binding domain, a DHHC-type zinc finger, six PHD-type zinc fingers, a SET domain, a post-SET domain, and a RING-type zinc finger. This protein is a member of the ASC-2/NCOA6 complex (ASCOM), which possesses histone methylation activity and is involved in transcriptional coactivation. SSTR5-AS1 increased the enrichment of H3K4me3 at the promoter region of SSTR5 by interacting with MLL3 and further induced the transcription of SSTR5.

Epithelial–mesenchymal transition (EMT) process widely occurred in the process of tumor metastasis. As the epithelial marker, E-cadherin was downregulated in numerous carcinomas and its inactivation was partly attributed to aberrant promoter hypermethylation [40]. In the present study, downregulation of E-cadherin and SSTR5-AS1 was detected in TGF-β-treated laryngeal carcinoma cells, indicating their essential role in LSCC metastasis. DNA methylation has been considered to be an extremely stable epigenetic marker until the identification of the TET gene family. The TET enzymes function as DNA demethylases which antagonize DNMT-mediated DNA methylation and gene repression. E-cadherin has been reported to be the TET target gene [22, 23], and in the present study, SSTR5-AS1 was proved to activate E-cadherin expression by recruiting TET1 to E-cadherin to hydrolyze 5′-mc to 5′-hmc. In summary, SSTR5-AS1 can cis-regulate the expression of SSTR5 by interacting with MLL3. In the process of laryngeal squamous cell carcinogenesis, when CpG sites hypermethylation occurs in the promoter region of E-cadherin, SSTR5-AS1 may also act as a tumor suppressor gene to upregulate the expression of E-cadherin by recruiting TET1 to E-cadherin to hydrolyze 5′-mc to 5′-hmc, thus inhibiting the occurrence of EMT.

## Conclusions

In conclusion, the present study demonstrates apparent differentially expressed mRNAs and lncRNAs profiles in metastatic LSCC tissues and corresponding normal tissues. SSTR5 may act as a tumor suppressor gene in LSCC, and aberrant DNA hypermethylation of the CpG sites clustered in the exon 1 and histone modification on its promoter region may be epigenetic mechanisms for its inactivation. SSTR5-AS1 may play anti-tumor role in LSCC and may be regulated by hypermethylation of the same CpG sites with SSTR5. SSTR5-AS1 inhibits laryngeal carcinoma cells proliferation, migration, and invasion. SSTR5-AS1 upregulates the expression of SSTR5 by interacting with MLL3. Furthermore, SSTR5-AS1 recruits TET1 to E-cadherin to hydrolyze 5′-mc to 5′-hmc and further activate E-cadherin expression.

## Methods

### Microarray processing and analysis

Agilent SBC Human (4*180 K) lncRNA Microarray (ID: 74348) was used to test the transcripts expression profiling. The microarray comprises 77103 lncRNAs and 18853 mRNAs, and the databases are from Gene bank, UCSC, LNCipedia, GENCODE v21/Ensemble, Lncrnadb, and Noncode. Four paired tissue specimens were obtained from metastatic LSCC patients with lymph node metastasis (Additional file 6: Table S1). Total RNA was extracted using TAKARA RNAiso Plus#9109 following the manufacturer’s instructions and checked for a RIN number to inspect RNA integrity by an Agilent Bioanalyzer 2100. Qualified total RNA was further purified by RNeasy Mini Kit (Cat#74106, QIAGEN, GmBH, Germany) and RNase-Free DNase Set (Cat#79254, QIAGEN, GmBH, Germany). The quality control of the LSCC tissues is listed in Additional file 7: Table S2. Total RNA was amplified and labeled by Low Input Quick Amp WT Labeling Kit (Cat.# 5190-2943, Agilent technologies, Santa Clara, CA, US), following the manufacturer’s instructions. Labeled cRNAs were purified by RNeasy mini kit (Cat.# 74106, QIAGEN, GmBH, Germany). Each slide was hybridized with 1.65 μg Cy3-labeled cRNA using Gene Expression Hybridization Kit (Cat.# 5188-5242, Agilent technologies, Santa Clara, CA, US), and after 17 h hybridization, slides were washed in staining dishes (Cat.# 121, Thermo Shandon, Waltham, MA, US) with Gene Expression Wash Buffer Kit (Cat.# 5188-5327, Agilent technologies, Santa Clara, CA, US). Slides were scanned by Agilent Microarray Scanner (Cat#G2565CA, Agilent technologies, Santa Clara, CA, US) with default settings. Data were extracted with Feature Extraction software 10.7 (Agilent technologies, Santa Clara, CA, US). Raw data were normalized by Quantile algorithm, limma packages in R.

Gene Ontology (GO) analysis and pathway analysis were used to find out the significant function and pathway of differentially expressed mRNAs in tumor tissues compared to corresponding normal tissues. According to the normalized signal intensity of specific expression in mRNAs and lncRNAs, mRNA-lncRNA expression correlation network was built to identify the correlations between mRNAs and lncRNAs. CeRNA network was constructed to discover ceRNA mechanism based on differentially expressed mRNAs and lncRNAs. The lncRNAs in cis- and in trans-targeted genes were also analyzed.

### Patients and specimens

The lncRNA expression identification, SSTR5 and SSTR5-AS1 expression were detected in 48 pairs of LSCC tissues and corresponding normal tissues. The 48 LSCC patients were from Otorhinolaryngology Head and Neck Surgery Biobank of Hebei Medical University, who received surgery between the years of 2016 and 2017. Written informed consent was obtained from all patients, and the study was approved by the Ethics Committee of Hebei Medical University. The patients consisted of 46 males and two females with a median age of 59.8 years (ranged from 41 to 73 years). Primary LSCC tissues and corresponding adjacent normal tissues were divided into two parallel parts: one part were formalin fixed and paraffin-embedded while the other part were frozen and stored at − 80 °C to extract genomic DNA and RNA. Information on clinical data and clinicopathologic characteristics was available from hospital recordings and pathological diagnosis and is listed in Additional file 8: Table S3.

### Cell culture and treatment

Human laryngeal carcinoma cell lines AMC-HN-8, TU212, TU686, and TU177 were purchased from American Type Culture Collection (ATCC, Manassas, VA, USA). Cells were detected and identified as mycoplasma and bacteria free according to ATCC’s instructions during the past 3 months. For drug treatment, the dose and timing of DNA methyltransferase inhibitor 5-Aza-dC and/or histone deacetylase (HDAC) inhibitor TSA were based on similar preliminary studies as well as published studies showing optimal reactivation of gene expression. Cells were seeded the day before the drug treatment. Cells (2 × 10^5^/mL) were treated with 5 μM 5-Aza-dC (Sigma, St Louis, MO, USA) for 72 h, and medium containing 5-Aza-dC was changed every 24 h, or with 0.3 μM TSA (Sigma, St Louis, MO, USA) for 24 h, or with the combination of 5 μM 5-Aza-dC for 48 h followed by 0.3 μM TSA for an additional 24 h. Control cells received no drug treatment. For TGF-β1 treatment, AMC-HN-8 cells were treated with 10 ng/ml of recombinant TGF-β1 (R&D Systems, Minneapolis, MN, USA) for 21 d with TGF-β1 replenishment every 2 days.

### RNA isolation and quantitative real-time RT-PCR assay

Total RNA was isolated from cell lines, frozen tumor, and corresponding normal tissues using TRIzol reagent (Invitrogen, Carlsbad, CA, USA). Two micrograms of RNA was employed to synthesize single-stranded cDNA using the advantage RT-for-PCR kit (Clontech, Palo Alto, CA, USA). Quantitative real-time RT-PCR analysis was performed with the cDNA from each sample as template, and Power SYBR Green PCR Master Mix (Life Technology, Foster City, CA, USA) was used as amplification reaction mixture. The GAPDH gene was used as an internal control. The 2^−ΔΔCT^ method was used to calculate fold change for the target genes [41]. The primers and reaction conditions for the genes detected in the present study are listed in Additional file 9: Table S4. All the samples were run in triplicate.

### Western blot analysis

Whole cell lysates from the cell lines were prepared by lysing the cells in ice-cold RIPA buffer. Total cell lysates were prepared, and 20 μg of proteins was subjected to Western blot analysis in 12% SDS-PAGE gels electrophoresis under denaturing and reducing conditions. Specific primary antibodies for SSTR5 (Rabbit antihuman polyclonal antibody, GTX79168, GeneTex, CA, USA) and E-cadherin, vimentin, CDH2, SNAI1, TWIST1, ZEB1 (Santa Cruz, San Diego, CA, USA) were used. To ensure equal loading in all the lanes, the blot was stripped and probed with antibody against GAPDH.

### Immunohistochemical staining

SSTR5 protein expression was determined by immunostaining using the avidin–biotin complex immunoperoxidase method. Rabbit antihuman polyclonal antibody for SSTR5 (1: 200 dilution; GTX79168, GeneTex, CA, USA) was employed to detect protein expression of SSTR5. Immunohistochemical staining was evaluated according to a scoring method reported previously [42]. Scoring accounted for both representation of the areas and intensities of the stains. All of the slides were reviewed concurrently by three experienced pathologists, who were blinded to the treatment factor.

### DNA extraction and sodium bisulfite treatment

Genomic DNA was extracted from laryngeal carcinoma cell lines, frozen LSCC tumor, and corresponding normal tissues using a simplified proteinase K digestion method. To examine the DNA methylation patterns, 1 µg of genomic DNA was bisulfite modified using Epitect Fast Bisulfite Conversion Kits (Qiagen, Germany) according to the manufacturer’s protocol. After the standard sodium bisulfite DNA modification, unmethylated cytosine residues were converted to thymine, whereas methylated cytosine residues were retained as cytosine at CpG sites.

### Methylated CpG sites distribution via bisulfite genomic sequencing (BGS) method

BGS assay was used to detect the distribution of methylated CpG sites of SSTR5 and SSTR5-AS1 in laryngeal carcinoma cell lines. The methylation status of the CpG sites of two regions according to the distribution of CpG islands of SSTR5 and SSTR5-AS1 was, respectively, detected: for SSTR5: promoter region: from − 311 to 27 bp, exon 1 region: from 7 to 1183 bp; for SSTR5-AS1: promoter region: from − 1231 to − 96 bp, exon 1 region: from − 71 to 331 bp. Fifty nanograms of bisulfite-modified DNA was subjected to PCR amplification, and the PCR products were cloned into pGEM-T vectors (Promega, Madison, WI, USA), and eight to ten clones of each specimen were sequenced by automated fluorescence-based DNA sequencing.

### Methylation analysis of the different regions via bisulfite conversion-specific and methylation-specific polymerase chain reaction (BS-MSP) assay

According to the distribution of the main methylated CpG sites by BGS analysis, the methylation status of the promoter and exon 1 regions of SSTR5 and SSTR5-AS1 was, respectively, analyzed by BS-MSP method: for SSTR5: promoter region: from − 191 to − 36 bp, exon 1 region: from 323 to 449 bp; for SSTR5-AS1: promoter region: from − 497 to − 372 bp, exon 1 region: from − 14 to 142 bp. The primers and reaction conditions are listed in Additional file 9: Table S4. BS-MSP products were analyzed on 2% agarose gel with ethidium bromide staining and were determined to have methylation if a visible band was observed in the methylation reaction. The reactions were performed in duplicate with each of the samples.

### Chromatin immunoprecipitation assay

The enrichment of the H3K4me3, H3K9ac, and H3K9me2 on the promoter region of SSTR5 in 5-Aza-dC-, TSA-, 5-Aza-dC/TSA-treated AMC-HN-8 cells, MLL3 and H3K4me3 on the promoter region of SSTR5 or TET1 on the promoter region of E-cadherin in SSTR5-AS1-transfected AMC-HN-8 cells was determined by chromatin immunoprecipitation (ChIP) assay with EZ-Magna ChIP A/G (17-10086, Upstate, Millipore, MA, USA) according to the manufacturer’s protocol. Antibodies against H3K4me3, H3K9ac, H3K9me2, MLL3, and TET1 (Upstate, Millipore, MA, USA) were used for immunoprecipitation. ChIP-derived DNA was quantified using real-time qPCR analysis. The primers are shown in Additional file 9: Table S4. Amplifications were performed in triplicate, and the enrichment was determined compared with input.

### Subcellular fractionation

To determine the cellular localization of SSTR5-AS1, cytosolic and nuclear fractions were collected from TU212 and TU686 cells using Nuclear/Cytosol Fractionation Kit (BioVision, Milpitas, CA, USA) in accordance with the manufacturer’s instructions. Then, RNA was isolated from the cytosolic and nuclear fractions, and the level of SSTR5-AS1 in cytoplasm and nucleus was determined by qRT-PCR method. GAPDH and U6 were, respectively, used as cytoplasmic and nuclear control.

### Cell transfection

For overexpression of SSTR5-AS1, the sequence of SSTR5-AS1 was synthesized and subcloned into pcDNA3.1 plasmid (Invitrogen, Carlsbad, CA, USA). The AMC-HN-8 and TU177 cells were transfected with SSTR5-AS1 expression plasmid (pcDNA3.1-SSTR5-AS1) or the empty vector (pcDNA3.1-NC) as control at a final concentration of 2 ug/uL using FuGENE HD Transfection Reagent (Promega, Madison, WI, USA). After selecting with G418, stable transfected cell lines were obtained. The coding sequences of MLL3 and TET1 were synthesized and subcloned into pcDNA3.1 plasmid. For inhibition of SSTR5-AS1, antisense oligodeoxynucleotides (ASO) specially targeted SSTR5-AS1 (ASO-SSTR5-AS1) (RiboBio, Guangzhou, China) was transfected into TU686 cells using Lipofectamine^®^2000 Reagent (Invitrogen, Carlsbad, CA, USA).

### Cell proliferation assay

The proliferation of cells was measured by cell-counting kit-8 (CCK-8) and clone formation assay. For CCK-8 assay, 10 μl of CCK8 (Dojindo, Japan) was added to the 100 ul cultured cells according to the manufacturer’s instruction, and after being incubated for 2 h, the absorbance of each well was measured at a wavelength of 492 nm. Proliferation rates were determined at 0, 24, 48, 72, 96 h after transfection. All experiments were repeated in triplicate. For clone formation assay, the transfected cells were regularly cultured for 1 week and then paraformaldehyde fixed and viola crystalline stained, counting clones under a microscope.

### Wound healing assay

For wound healing assay, a wound was made by a straight scratch with a 200-μL pipette tip in the cultured cells, and then the images were captured at the same position of each well for 0 h, 12 h, and 24 h after the wound was created under a microscope. The relative distance of cell migration to the scratched area was measured, and a healing percentage was calculated. The experiment was performed in triplicate.

### Cell invasion assay

The invasiveness of cells was evaluated in 24-well transwell chambers (Corning, Kennebunk, ME, USA). The number of cells invaded through the membrane to the lower surface was counted in five microscopic fields (at × 100 magnification) per filter. The experiments were performed in triplicate.

### RNA immunoprecipitation assay

The binding of SSTR5-AS1 to MLL3 or TET1 was detected by RNA immunoprecipitation method using MLL3 or TET1 antibody (Upstate, Millipore, MA, USA) and the Magna RIP™ RNA-Binding Protein Immunoprecipitation Kit (Millipore, Billerica, MA, USA) according to the manufacturer’s instructions. The IgG antibody was used as negative control. The purified RNA was subjected to qRT-PCR analysis.

### hMeDIP-qPCR analysis

Genomic DNA from cells was prepared using a phenol–chloroform method. The hMeDIP assay was performed as previously described [43]. Briefly, genomic DNA was denatured and then immunoprecipitated with anti-5hmC antibody or IgG control antibody and protein G magnetic dynabeads (Invitrogen, Carlsbad, CA, USA). The pulled-down DNA was analyzed by qPCR.

### Statistical analysis

Statistical analysis was performed with SPSS19.0 software package (SPSS Company, Chicago, Illinois, USA). The real-time RT-PCR results were expressed as the mean ± S.D. Student’s t test was used to compare the expression means between different groups. The status of gene methylation between different groups was analyzed using Pearson’s Chi-square test. All statistical tests were two sided, and *P* < 0.05 was considered to be statistically significant.

## Additional files

**Additional file 1: Table S5.** The probe and expression of SSTR5 and SSTR5-AS1 in microarray assay.

**Additional file 2: Table S6.** Protein expression and methylation status of SSTR5 in LSCC tumor tissues and corresponding normal tissues.

**Additional file 3: Table S7.** Methylation status of SSTR5-AS1 in LSCC tumor tissues and corresponding normal tissues.

**Additional file 4: Table S8.** The correlation between the protein expression and methylation status of SSTR5 in LSCC tumor tissues.

**Additional file 5: Fig. S1.** The influence of downregulation of SSTR5-AS1 on laryngeal carcinoma cells proliferation, migration, and invasion. A. Downregulation of SSTR5-AS1 was detected by qRT-PCR in ASO-transfected TU686 cells. * P < 0.05. B. Knockdown of SSTR5-AS1 increased TU686 cells proliferation. * P < 0.05. C. Knockdown of SSTR5-AS1 increased TU686 cells migration detected by wound healing assay. * P < 0.05. D. Knockdown of SSTR5-AS1 increased TU686 cells invasiveness detected by transwell invasion assay. * P < 0.05.

**Additional file 6: Table S1.** The clinical pathological characteristics of the four LSCC cases for microarray assay.

**Additional file 7: Table S2.** The quality control of the LSCC tissues for microarray assay.

**Additional file 8: Table S3.** Clinicopathologic characteristics of LSCC cases.

**Additional file 9: Table S4.** Primer sequences and reaction conditions of the genes in this study.

## Data Availability

The datasets used and/or analyzed during the current study are available from the corresponding author on reasonable request.

## Abbreviations

LSCC: laryngeal squamous cell carcinoma
lncRNAs: long noncoding RNAs
HNSC: head and neck squamous cell carcinoma
GO: Gene Ontology
ACC: adrenocortical carcinoma
KIRC: kidney renal clear cell carcinoma
PCPG: pheochromocytoma and paraganglioma
5′-mc: 5′-methylcytosine
5′-hmc: 5′-hydroxymethylcytosine
EMT: epithelial–mesenchymal transition
BGS: bisulfite genomic sequencing
BS-MSP: bisulfite conversion-specific and methylation-specific polymerase chain reaction
ChIP: chromatin immunoprecipitation
CCK-8: cell-counting kit-8
