# Supplementary material for: Aberrant methylation-mediated downregulation of lncRNA SSTR5-AS1 promotes progression and metastasis of laryngeal squamous cell carcinoma
Source: Epigenetics Chromatin. 2019 Jun 13;12:35. doi: 10.1186/s13072-019-0283-8 (PMC6563380; doi:10.1186/s13072-019-0283-8)
Supplement: Supplementary file 1 — Additional file 1: Table S5. The probe and expression of SSTR5 and SSTR5-AS1 in microarray assay. [file 13072_2019_283_MOESM1_ESM.docx]

Table S5: The probe and expression of SSTR5 and SSTR5-AS1 in microarray assay

| Probe name | P | Fold  change | Probe  Seq | Gene Symbol | Accession |
| --- | --- | --- | --- | --- | --- |
| CUST_134354_PI430048170 | 0.011 | 0.06 | GCAGTGAGGACACGCGTGTTTGACAACTGCTCCCCTGAATAAATGCGAGGATAAATGTTT | SSTR5 | NM_001053 |
| CUST_28643_PI430048170 | 0.026 | 0.03 | GGAGCCATTTTCCCTGATTTGTTTGCCTCATCAACTTTTCAATCAATAAAAAAGTGCATT | SSTR5-AS1 | NR_027242 |
