# Supplementary material for: Aberrant methylation-mediated downregulation of lncRNA SSTR5-AS1 promotes progression and metastasis of laryngeal squamous cell carcinoma
Source: Epigenetics Chromatin. 2019 Jun 13;12:35. doi: 10.1186/s13072-019-0283-8 (PMC6563380; doi:10.1186/s13072-019-0283-8)
Supplement: Supplementary file 2 — Additional file 2: Table S6. Protein expression and methylation status of SSTR5 in LSCC tumor tissues and corresponding normal tissues. [file 13072_2019_283_MOESM2_ESM.docx]

Table S6: Protein expression and methylation status of SSTR5 in LSCC tumor tissues and corresponding normal tissues

| Groups | N | Protein expression | | Methylation frequency  (Promoter) | | Methylation frequency  (Exon 1) | |
| --- | --- | --- | --- | --- | --- | --- | --- |
|  |  | n (%) | P | n (%) | P | n (%) | P |
| Normal tissues | 48 | 35(72.9) |  | 5(10.4) |  | 6(12.5) |  |
| Tumor tissues | 48 | 16(33.3) | <0.001 | 9(18.7) | 0.247 | 27(56.3) | <0.001 |
