# Supplementary material for: Aberrant methylation-mediated downregulation of lncRNA SSTR5-AS1 promotes progression and metastasis of laryngeal squamous cell carcinoma
Source: Epigenetics Chromatin. 2019 Jun 13;12:35. doi: 10.1186/s13072-019-0283-8 (PMC6563380; doi:10.1186/s13072-019-0283-8)
Supplement: Supplementary file 3 — Additional file 3: Table S7. Methylation status of SSTR5-AS1 in LSCC tumor tissues and corresponding normal tissues. [file 13072_2019_283_MOESM3_ESM.docx]

Table S7: Methylation status of SSTR5-AS1 in LSCC tumor tissues and corresponding normal tissues

| Group | N | Methylation frequency | | | |
| --- | --- | --- | --- | --- | --- |
|  |  | Promoter | | Exon 1 | |
|  |  | n (%) | P | n (%) | P |
| Normal tissues | 48 | 7(14.6) |  | 6(12.5) |  |
| Tumor tissues | 48 | 25(52.1) | <0.001 | 8(16.7) | 0.563 |
