# Supplementary material for: Aberrant methylation-mediated downregulation of lncRNA SSTR5-AS1 promotes progression and metastasis of laryngeal squamous cell carcinoma
Source: Epigenetics Chromatin. 2019 Jun 13;12:35. doi: 10.1186/s13072-019-0283-8 (PMC6563380; doi:10.1186/s13072-019-0283-8)
Supplement: Supplementary file 4 — Additional file 4: Table S8. The correlation between the protein expression and methylation status of SSTR5 in LSCC tumor tissues. [file 13072_2019_283_MOESM4_ESM.docx]

Table S8: The correlation between the protein expression and methylation status of SSTR5 in LSCC tumor tissues

| Methylation status | Protein expression | | P |
| --- | --- | --- | --- |
|  | Positive | Negative |  |
| Promoter |  |  |  |
| Methylation | 2 | 7 |  |
| Unmethylation | 14 | 25 | 0.433 |
| Exon 1 |  |  |  |
| Methylation | 4 | 23 |  |
| Unmethylation | 12 | 9 | 0.002 |
