# Supplementary material for: Aberrant methylation-mediated downregulation of lncRNA SSTR5-AS1 promotes progression and metastasis of laryngeal squamous cell carcinoma
Source: Epigenetics Chromatin. 2019 Jun 13;12:35. doi: 10.1186/s13072-019-0283-8 (PMC6563380; doi:10.1186/s13072-019-0283-8)

Fig. S1: The influence of down regulation of SSTR5-AS1 on laryngeal carcinoma cells proliferation, migration, and invasion

A. Down-regulation of SSTR5-AS1 was detected by qRT-PCR in ASO transfected TU686 cells. * P < 0.05. B. Knockdown of SSTR5-AS1 increased TU686 cells proliferation. * P < 0.05. C. Knockdown of SSTR5-AS1 increased TU686 cells migration detected by wound healing assay. * P < 0.05. D. Knockdown of SSTR5-AS1 increased TU686 cells invasiveness detected by transwell invasion assay. * P < 0.05.


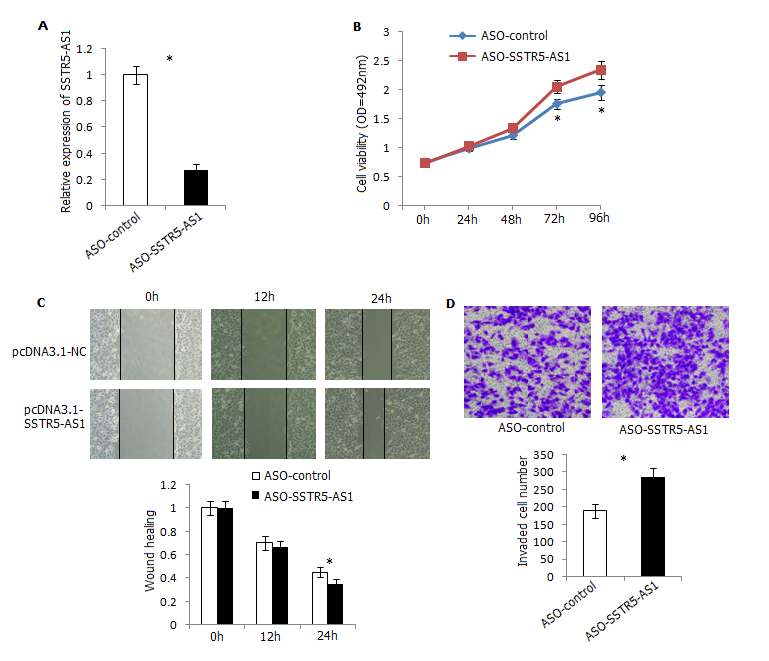

Supplement: Supplementary file 5 — Additional file 5: Fig. S1. The influence of downregulation of SSTR5-AS1 on laryngeal carcinoma cells proliferation, migration, and invasion. A. Downregulation of SSTR5-AS1 was detected by qRT-PCR in ASO-transfected TU686 cells. * P < 0.05. B. Knockdown of SSTR5-AS1 increased TU686 cells proliferation. * P < 0.05. C. Knockdown of SSTR5-AS1 increased TU686 cells migration detected by wound healing assay. * P < 0.05. D. Knockdown of SSTR5-AS1 increased TU686 cells invasiveness detected by transwell invasion assay. * P < 0.05. [file 13072_2019_283_MOESM5_ESM.docx]
