# Supplementary material for: Aberrant methylation-mediated downregulation of lncRNA SSTR5-AS1 promotes progression and metastasis of laryngeal squamous cell carcinoma
Source: Epigenetics Chromatin. 2019 Jun 13;12:35. doi: 10.1186/s13072-019-0283-8 (PMC6563380; doi:10.1186/s13072-019-0283-8)
Supplement: Supplementary file 6 — Additional file 6: Table S1. The clinical pathological characteristics of the four LSCC cases for microarray assay. [file 13072_2019_283_MOESM6_ESM.docx]

Table S1: The clinical pathological characteristics of the 4 LSCC cases for microarray assay

| Sample | Gender | Age | pathologic type | TNM stage |
| --- | --- | --- | --- | --- |
| 1 | Male | 64 | laryngeal squamous cell carcinoma | T1N2M0 |
| 2 | Male | 54 | laryngeal squamous cell carcinoma | T1N2M0 |
| 3 | Male | 65 | laryngeal squamous cell carcinoma | T1N2M0 |
| 4 | Male | 57 | laryngeal squamous cell carcinoma | T4N2M0 |
