# Supplementary material for: Aberrant methylation-mediated downregulation of lncRNA SSTR5-AS1 promotes progression and metastasis of laryngeal squamous cell carcinoma
Source: Epigenetics Chromatin. 2019 Jun 13;12:35. doi: 10.1186/s13072-019-0283-8 (PMC6563380; doi:10.1186/s13072-019-0283-8)
Supplement: Supplementary file 7 — Additional file 7: Table S2. The quality control of the LSCC tissues for microarray assay. [file 13072_2019_283_MOESM7_ESM.docx]

Table S2: The quality control of the LSCC tissues for microarray assay

| Sample | Concentration  (μg/μL) | A260/A280 | RIN | 28S/18S |
| --- | --- | --- | --- | --- |
| 1C | 677 | 2.01 | 7.2 | 1.1 |
| 1N | 230 | 1.92 | 7.2 | 0.7 |
| 2C | 382 | 1.92 | 7.0 | 0.7 |
| 2N | 164 | 1.85 | 7.1 | 0.9 |
| 3C | 344 | 1.96 | 7.8 | 1.3 |
| 3N | 203 | 1.88 | 7.0 | 0.8 |
| 4C | 466 | 2.03 | 8.4 | 1.5 |
| 4N | 74 | 1.92 | 7.7 | 1.1 |
