# Supplementary material for: Aberrant methylation-mediated downregulation of lncRNA SSTR5-AS1 promotes progression and metastasis of laryngeal squamous cell carcinoma
Source: Epigenetics Chromatin. 2019 Jun 13;12:35. doi: 10.1186/s13072-019-0283-8 (PMC6563380; doi:10.1186/s13072-019-0283-8)
Supplement: Supplementary file 8 — Additional file 8: Table S3. Clinicopathologic characteristics of LSCC cases. [file 13072_2019_283_MOESM8_ESM.docx]

Table S3: Clinicopathologic characteristics of LSCC cases

| Groups | N (%) |
| --- | --- |
| Age |  |
| ＜60 | 25(52.1) |
| ≥60 | 23(47.9) |
| Gender |  |
| Male | 46(95.8) |
| Female | 2(4.2) |
| Smoking |  |
| Negative | 10(20.8) |
| Positive | 38(79.2) |
| TNM stage |  |
| Ⅰ | 3(6.3) |
| Ⅱ | 16(33.3) |
| Ⅲ | 17(35.4) |
| Ⅳ | 12(25.0) |
| Pathological differentiation of tumor |  |
| Well | 20(41.7) |
| Moderate | 16(33.3) |
| Poor | 12(25.0) |
| LN metastasis |  |
| negative (N0) | 22(45.8) |
| positive (N1/2/3) | 26(54.2) |
