# Supplementary material for: Aberrant methylation-mediated downregulation of lncRNA SSTR5-AS1 promotes progression and metastasis of laryngeal squamous cell carcinoma
Source: Epigenetics Chromatin. 2019 Jun 13;12:35. doi: 10.1186/s13072-019-0283-8 (PMC6563380; doi:10.1186/s13072-019-0283-8)
Supplement: Supplementary file 9 — Additional file 9: Table S4. Primer sequences and reaction conditions of the genes in this study. [file 13072_2019_283_MOESM9_ESM.docx]

Table S4: Primer sequences and reaction conditions of the genes in this study

| PCR types | Gene | Primers | Annealing temperature (℃) | Product size (bp) |
| --- | --- | --- | --- | --- |
| RT-PCR | SSTR5 | F:5’-GTCAACATCGTCAACCTGGC-3’ |  |  |
|  |  | R:5’-GGCGGAAGTTGTCAGAGAGG-3’ | 62 | 139 |
|  | SSTR5-AS1 | F:5'-ACTACAGGTGCCATCAGACC-3' |  |  |
|  |  | R:5'-AGCCTGCCATCCTAACACTT-3' | 61 | 146 |
|  | HOTAIR | F:5’-AGGCAAATGTCAGAGGGTT-3’ |  |  |
|  |  | R:5’-CTTAAATTGGGCTGGGTC-3’ | 55 | 199 |
|  | TINCR | F:5’-TCCCAGGTGGACCATGAAAC-3’ |  |  |
|  |  | R:5’-ACAGCAGAGCTGAAAGGCTC-3’ | 55 | 152 |
|  | LINC00511 | F:5’ -GGGCGACTACTGTTACCTCG-3’ |  |  |
|  |  | R:5’-TCCTCACCACGTCCAAACAG-3’ | 56 | 104 |
|  | LINC00520 | F:5’-CCATTGAAGACCGTAACAC-3’ |  |  |
|  |  | R:5’-GAAGTAGAGTAGCCAGGATT-3’ | 57 | 103 |
|  | MEG3 | F:5’-TACACCTCACGAGGGCACTA-3’ |  |  |
|  |  | R:5’-CAGGGCTTAATGCCCAATGC-3’ | 58 | 187 |
|  | ZNF667-AS1 | F:5’-CATCACTACCATCCATCACTA-3’ |  |  |
|  |  | R:5’-CCAGGCAGAGAAGGATAA-3’ | 57 | 186 |
|  | HULC | F:5’-CAGACCAAAGCATCAAGCAAGA-3’ |  |  |
|  |  | R:5’-ACAAATTTGCCACAGGTTGAACA-3’ | 54 | 100 |
|  | E-cadherin | F:5’-CGAGAGCTACACGTTCACGG-3 |  |  |
|  |  | R:5’-GGCCTTTTGACTGTAATCACACC-3' | 59 | 162 |
|  | Vimentin | F:5’-CGCCTGCAGGATGAGATTCAG-3’ |  |  |
|  |  | R:5’-TCAGGGAGGAAAAGTTTGGAAGA-3’ | 58 | 175 |
|  | CDH2 | F:5’-CAACTTGCCAGAAAACTCCAGG-3’ |  |  |
|  |  | R:5’-ATGAAACCGGGCTATCTGCTC-3' | 59 | 205 |
|  | SNAI1 | F:5’-ACGAGGTGTGACTAACTAT-3’ |  |  |
|  |  | R:5’-CGACAAGTGACAGCCATT-3’ | 58 | 197 |
|  | TWIST1 | F:5’-ACCATCCTCACACCTCTG-3’ |  |  |
|  |  | R:5’-GATTGGCACGACCTCTTG-3’ | 60 | 132 |
|  | ZEB1 | F:5’-TCATCGCTACTCCTACTGT-3’ |  |  |
|  |  | R:5’-TCACTGTCTTCATCCTCTTC-3’ | 54 | 171 |
|  | GAPDH | F:5’-AGGTGAAGGTCGGAGTCAACG-3’ |  |  |
|  |  | R:5’-AGGGGTCATTGATGGCAACA-3’ |  | 104 |
| BGS | SSTR5 |  |  |  |
|  | Promoter | F:5’-GGTAGTAGTATTGTAGGGTAG-3’ |  |  |
|  |  | R:5’-AATCCCTAAACCAACACATAC-3’ | 57 | 338 |
|  | Exon 1 | F:5’-GTATGTGTTGGTTTAGGGATTTA-3’ |  |  |
|  |  | R:5’-TCACAACTTACTAATCTACATAAAC-3’ | 55 | 1177 |
|  | SSTR5-AS1 |  |  |  |
|  | Promoter | F:5’-TATAGTTTGTTGGTTTGTATAAG-3’ |  |  |
|  |  | R:5’-CTTCTTCTCTTACAAAACCTA-3’ | 56 | 1136 |
|  | Exon 1 | F:5’-TTGAATTAGTATATGTAGGTA-3’ |  |  |
|  |  | R:5’-TACCACCTCTCCCTATATATC-3’ | 55 | 402 |
| BS-MSP | SSTR5 |  |  |  |
|  | Promoter |  |  |  |
|  | First-step | F:5’-GGTAGTAGTATTGTAGGGTAG-3’ |  |  |
|  |  | R:5’-AATCCCTAAACCAACACATAC-3’ | 57 | 338 |
|  | Second-step |  |  |  |
|  | Methylation | F: 5'-GTTGTCGTTATATCGATAGTAGTCG-3' |  |  |
|  |  | R: 5'-AACGCGAAATCACCAAATAAACACG-3' | 56 | 156 |
|  | Unmethylation | F: 5'-GTTGTTGTTATATTGATAGTAGTTG-3' |  |  |
|  |  | R: 5'-AACACAAAATCACCAAATAAACACA-3' | 56 | 156 |
|  | Exon 1 |  |  |  |
|  | First-step | F:5’-GTATGTGTTGGTTTAGGGATTTA-3’ |  |  |
|  |  | R:5’-AATACACCACTACCAAATAAC-3’ | 55 | 512 |
|  | Second-step |  |  |  |
|  | Methylation | 5'-ATTTTTAATTTGGTAGTGGTCGACG-3' |  |  |
|  |  | 5'-CGCCGTCCAACGTCATAACCAAACG-3' | 57 | 127 |
|  | Unmethylation | 5'-ATTTTTAATTTGGTAGTGGTTGATG-3' |  |  |
|  |  | 5'-CACCATCCAACATCATAACCAAACA-3' | 57 | 127 |
|  | SSTR5-AS1 |  |  |  |
|  | Promoter |  |  |  |
|  | First-step | F:5’- AGAAGATATTGGTGAATTGGT-3’ |  |  |
|  |  | R:5’-CTTCTTCTCTTACAAAACCTA-3’ | 56 | 427 |
|  | Second-step |  |  |  |
|  | Methylation | F:5'-GTCGTTTAGCGTTATGATTAGGCG-3' |  |  |
|  |  | R:5'-ATTCTCAACCTAACAATAACCGACG-3' | 57 | 126 |
|  | Unmethylation | F:5'-GTTGTTTAGTGTTATGATTAGGTG-3' |  |  |
|  |  | R:5'-ATTCTCAACCTAACAATAACCAACA-3' | 57 | 126 |
|  | Exon 1 |  |  |  |
|  | First-step | F:5’-TTGAATTAGTATATGTAGGTA-3’ |  |  |
|  |  | R:5’-TACCACCTCTCCCTATATATC-3’ | 55 | 402 |
|  | Second-step |  |  |  |
|  | Methylation | F:5’-GGCGCGGGATTATTGGGTAAATACG-3’ |  |  |
|  |  | R:5’-ACTATCGCCATATCGACAACAACCG-3’ | 57 | 156 |
|  | Unmethylation | F:5’-GGTGTGGGATTATTGGGTAAATATG-3’ |  |  |
|  |  | R:5’-ACTATCACCATATCAACAACAACCA-3’ | 57 | 156 |
| ChIP | SSTR5 | F:5'-TGTCGCCATATCGACAGCAG-3' |  |  |
|  |  | R:5'-TGTTTAGCTTGGCCGGGAAT-3' | 58 | 121 |
|  | E-cadherin | F:5'- ACCGCGTCTATGCGAGGCCG-3' |  |  |
|  |  | R:5'- CCGTACCGCTGATTGGCTGA-3' | 56 | 113 |

F: Forward primer; R: Reverse primer.

|  |
| --- |
|  |
